# Enhanced Isolation of *Brucella abortus* from Lymphoid Tissues of Mice Orally Infected with Low Doses in a Two-Step Procedure

**DOI:** 10.3390/microorganisms13071442

**Published:** 2025-06-20

**Authors:** Ana Beatriz Sánchez-Argáez, Estefania Herrera-Torres, Martha Cecilia Moreno-Lafont, Leopoldo Flores-Romo, Rubén López-Santiago

**Affiliations:** 1 Departamento de Inmunología, Escuela Nacional de Ciencias Biológicas, Instituto Politécnico Nacional, Prol. de Carpio y Plan de Ayala s/n, Del. Miguel Hidalgo, Ciudad de México 11340, Mexico; ana.sanchez@cinvestav.mx (A.B.S.-A.); estefa_h@hotmail.com (E.H.-T.); mlafont@ipn.mx (M.C.M.-L.); 2 Departamento de Biomedicina Molecular y Departamento de Biología Celular, Centro de Investigación y de Estudios Avanzados (CINVESTAV), Instituto Politécnico Nacional, Av. IPN No. 2508. Del. Gustavo A. Madero, Ciudad de México 07360, Mexico; leflores@cinvestav.mx

**Keywords:** *Brucella abortus*, oral infection, mouse models, isolation of *Brucella*

## Abstract

The main aspects of brucellosis have been studied in animal models to better understand the pathogenesis of the disease. Mice are the most common animal model of brucellosis. To verify that the infection has been successfully induced, it is necessary to assess the presence of *Brucella* in experimentally infected mice. Traditionally, high doses of *Brucella* have been used to establish detectable infection in oral murine models but prevent the emulation of natural pathogenesis. We propose the use of a low dose (1 × 10^6^ CFUs) to establish a more realistic oral infection model. Using a two-step procedure consisting of selective broth enrichment followed by agar isolation, we were able to recover bacteria from gut-associated lymphoid tissues (mesenteric lymph nodes and Peyer’s patches), the spleen, and feces during the early and late stages of infection (1 h and up to 5 weeks). This technique promotes the study of early infection stages and systemic dissemination without the need for high doses to induce infection orally. It also demonstrates that *Brucella* remains in the intestinal-associated lymphoid tissues at time points when the infection is already systemically established.

## 1. Introduction

Brucellosis is a disease that develops in mammals, including humans, and is caused by bacteria of the genus *Brucella*. It is considered an important anthropozoonosis spread worldwide. Some authors have suggested that new human cases currently reach 1.6–2.1 million per year, an alarming number when compared with the 500,000 cases frequently reported [1,2]. Humans acquire the infection by consuming or handling products derived from infected animals. Brucellosis remains a health problem in Latin America, China, Russia, and the Middle East, even though it has been successfully eradicated in cattle and, therefore, in humans from the United States, Canada, Japan, and New Zealand, among others. However, a lack of sanitation control and an increase in immigrants carrying the disease puts countries that have eradicated brucellosis at risk again [3]. The pathophysiology of brucellosis is poorly described, and there is no consensus on an adequate classification of its clinical course. Therefore, a more detailed and precise description of brucellosis is of great importance. In scientific research, the use of animal models is required to study human diseases, including brucellosis. In the *Brucella* infection model, the isolation of the pathogen is vital to confirming successful infection, regardless of the administration route of the bacteria. Reviewing the literature, we found that, for many years, animal models of brucellosis had been induced by other ways rather than the natural infection pathway. These models had induced systemic infection, usually inoculating the bacteria intravenously or intraperitoneally, which not only made it easier to recover the bacteria by classic methods, such as microbiological isolation, but also evaded important natural defense mechanisms of the host [4,5]. To better comprehend the development of the disease, it is essential that an animal model simulates a natural infection to mimic the natural bacterial entry and bacterial load that cause the disease. Nonetheless, oral inoculation models mimicking this route appear to require higher doses of *Brucella*, since the bacteria are subjected to several barriers in the gastrointestinal tract. That is possibly one of the reasons why other research groups employ high doses of inoculation, ranging from 1 × 10^9^ to 2 × 10^10^ CFUs [6,7]. Therefore, in this work we propose a two-step method that allows the enrichment of a small bacterial load of *Brucella abortus* in a selective broth medium that can be recovered from lymphoid and non-lymphoid tissues for subsequent agar isolation. To do so, we used a murine model of infection with low doses of bacteria administered through the natural oral entryway.

## 2. Materials and Methods

### 2.1. Mice and Infection

To induce the infection, BALB/c mice aged 4–6 weeks were orally inoculated with a stainless-steel curved feeding needle—size 20 G, L × diam. 1.5 in. × 2.25 mm, ball (Sigma-Aldrich, St. Louis, MO, USA). The experiments in this work followed the ARRIVE guidelines and the National Institute of Health Guide for the Care and Use of Laboratory Animals (NIH Publications No. 8023, revised 1978).

The animals received 100 µL sodium bicarbonate (0.35 M, Técnica Química, Mexico) to buffer the stomach pH 15 min prior to the infection with *Brucella*. The mice were orally administered 100 μL 1 × 10^6^, 5 × 10^6^, or 10 × 10^6^ colony-forming units (CFUs) of *B. abortus* strain 2308, originally described in 1940 as a virulent strain recovered from an aborted fetus of a cow that had been in contact with cattle experimentally infected to induce infection [8]. The bacterial suspensions were adjusted to 0.4 OD at 540 nm (Spectra 20; Bausch and Lomb, Laval, QC, Canada) in injectable water. The CFUs of the *B. abortus* were confirmed by plating tenfold serial dilutions of the cultures on TSA.

### 2.2. Tissue Samples

Ten groups of three mice each were sacrificed at different time points ranging from 1 h up to 5 weeks postinfection. Peyer’s patches (PPs), mesenteric lymph nodes (MLNs), and spleens were removed and weighed, and each tissue was mechanically homogenized in 1 mL cold sterile PBS (Sigma-Aldrich, St. Louis, MO, USA). When needed, 100 µL each of the cell suspension was used for serial dilutions, and the remaining 900 μL was reserved for the selective broth-culture method.

### 2.3. Bacteria Isolation by Direct Plating

Using 100 μL cell suspension obtained from each tissue, 10^−1^, 10^−2^, and 10^−3^ dilutions were made with sterile distilled water to lyse the cells and release the intracellular bacteria. Then, 100 µL of each dilution was plated in duplicate on plates with trypticase soy agar (TSA) (BD Bioxon, Mexico City, Mexico) supplemented with Modified *Brucella* Selective Supplement (reconstituted as indicated by manufacturer) (Oxoid™; Thermo Fisher, Waltham, MA, USA) and incubated at 37 °C in 5% CO_2_ for 48 h. The growth of the CFUs was monitored for up to 2 weeks. The number of CFUs was calculated by applying the following formula: CFU/mL = (No. CFU × inverse of dilution)/volume of plated suspension.

### 2.4. Bacteria Isolation by Selective Broth Medium Enrichment

The remaining 900 µL cell suspensions from each sample were placed in independent conical tubes (15 mL, Falcon™; Corning, Somerville, MA, USA) with 3 mL trypticase soy broth (TSB) supplemented with Modified *Brucella* Selective Supplement (Oxoid™; Thermo Fisher, Waltham, MA, USA). The tubes were incubated at 37 °C for 72 h in an orbital shaker at 150–180 rpm (Barnstead Lab-line Max^Q^ 4000, Marshall Scientific, Hampton, NH, USA).

### 2.5. Collection of Fecal Samples and Bacteria Isolation

Six additional mice were used to obtain fecal samples. The mice were divided into two groups (control and infected) and were administered injectable water or 5 × 10^6^ CFUs *B. abortus* 2308. The feces were recollected from 1 h up to 5 weeks postadministration, weighted to obtain approximately 0.1 g. Subsequently, they were homogenized with 2 mL TSB supplemented with Oxoid™ Modified *Brucella* Selective Supplement and strained through a cell strainer (40 mm; Corning, Somerville, MA, USA) to eliminate any remaining large particles. Once strained, 70 μL suspension was placed in conical tubes containing 3.93 mL TSA supplemented with Modified *Brucella* Selective Supplement (30 μL/mL) (Oxoid, Thermo Fisher, Waltham, MA, USA). The tubes were incubated at 37 °C for 72 h in an orbital shaker at 150–180 rpm. Finally, 20 mL of each tube was transferred to TSA (BD Bioxon, Mexico City, Mexico) plates and incubated at 37 °C for 48 h.

## 3. Results

First, we sought to demonstrate that *Brucella* could be recovered even when using lower doses for infection. To do this, different groups of mice were inoculated with the selected doses of the bacteria. Using the proposed method, the bacteria could not be recovered from the mice infected with the lowest dose of *B. abortus* 2308 (1 × 10^6^ CFUs). However, we were able to recover the bacteria from the mice infected with 5 × 10^6^ and 10 × 10^6^ CFUs in all the organs tested with the selective culture broth (Table 1). Based on these results, the dose of 5 × 10^6^ CFUs was chosen for the subsequent experiments.

Once it was demonstrated that the bacteria could be recovered even at low doses, we attempted recovery using the common TSA plaque-growth technique. The bacterial growth (reported as CFUs) was inconsistent between the different tissues and mice from the same group, and it was only observed at certain time points through kinetics. These results were not consistent and repeatable in three additional experiments performed (Table 2).

Since it was not possible to recover the *Brucella* following the classic direct plating technique, we decided to use the selective culture broth enrichment technique and analyze the progression of the infection at different time points, ranging from the early stages to the acute and chronic phases. Using this technique, the bacteria was recovered as early as 1 h postinfection from the PPs and MLNs, and, from the feces, 2 h postinfection. In the spleen, they were detected 72 h after infection, indicating that the *Brucella* disseminated systemically. The results obtained from these experiments demonstrate that *Brucella* is not only capable of breaching the intestinal barrier and remaining both in PPs and MLNs, but it is also being released in feces during shedding. These findings show that the bacterium is maintained in gut-associated lymphoid organs, at least during the follow-up time of this study, which could be one of the reasons why it can be isolated from feces. Another reason could be that it harbors epithelial cells, or it is even in the intestinal lumen. These last two possibilities are part of ongoing research. Interestingly, the bacteria were not detectable after a week of infection in the PPs, feces, and spleens but remained constant in the MLNs throughout the shedding kinetics. The presence of the *Brucella* was monitored for up to 5 weeks, corresponding to a chronic infection. The results shown in Table 3 correspond to three repetitions of each time postinfection.

## 4. Discussion

Due to its zoonotic potential, *Brucella abortus* is one of the most common causes of human brucellosis, with oral and inhalation intake being the most common ways of infection. With an infective dose ranging from 10 to 100 bacteria and a lack of prophylactic treatments, the infection may last for years [9]. A successful and more realistic *Brucella* animal model would be one induced by the administration of *Brucella* orally or intragastrically. Different research groups have tried to establish models of oral infection, but among the difficulties they have found is the need to administer high doses of *Brucella* (≥10^10^ CFUs) to detect viable bacteria in mouse organs [10]. The need for these high doses might be related to the hostile environments the bacteria confront, such as the acid pH of the stomach, the epithelial barrier of the intestine, competition with the mouse microbiota, and other factors, including the molecules and cells of the intestinal immune system [11,12,13,14]. In an oral model of infection or when natural infections occur, the bacteria are subjected to all of the above barriers. Consequently, the number of bacteria that can overcome these barriers must be minimal, and the bacterial load established in the organs is lower than that required to recover *Brucella*, making the isolation difficult to carry out by the usual methods. In this work, the murine infection model was implemented with a dose of 5 × 10^6^ CFUs, which is considered a low dose: between 200 and 4000 times lower than the doses reported elsewhere. The technique implemented in this work aimed at detecting the low bacterial count that survived the mechanisms of innate immunity. The selective medium is necessary when attempting to culture tissues that are in constant contact with other antigens, as in the case of mucosa-associated lymphoid tissue. Selective media for *Brucella* increase the successful isolation of the bacteria. They have been proposed and used due to their high sensitivity, as confirmed by our results. Even though molecular biology techniques such as PCR are more sensitive, they also increase the complexity of the assays because of the need for special equipment, specially trained personnel, and expensive reagents [15].

The development of a model of *Brucella*’s natural entryway will allow us to study its invasive behavior and follow its pathway in the tissues for the bacteria upon entry. These tissues include the intestinal epithelium, as well as the gut-associated lymphoid tissues of entry, as this is where the immune response is initiated. Rungue et al. reported that orally inoculated *Brucella melitensis* produced the greatest damage to the epithelial barrier after 72 h, causing increased intestinal permeability, histopathological damage, and inflammation [10]. We reported the presence of *Brucella* within 1 h after inoculation, indicating that *Brucella* was able to cross the physical and chemical barriers of the gastrointestinal tract and successfully arrive at the intestine.

Rungue et al. also suggested that increased permeability favors the dissemination of the bacteria to other tissues, such as the liver. Our results agree with those of Rungue et al., since in our model, it was possible to detect the bacteria in the spleen after 72 h and it was possible to recover it even at five weeks [10]. 

The persistence of *Brucella* in gut-associated tissues for long periods, as we report here, have led us to propose that the gut should be studied more thoroughly to better understand the pathophysiology of brucellosis. Von Bargen et al. have proposed the cervical lymph node as a reservoir for *Brucella*. We do not know if other lymphoid tissues, such as the MLNs and the PPs, are also reservoirs. This has not been reported because the bacteria cannot be detected with routine techniques [7]. These tissues are very small; for example, a PP has a diameter of approximately 1 to 2 mm, which further complicates bacterial detection. However, our enrichment technique overcomes these limitations [16].

With the technique we used, it was possible to detect the presence of *Brucella* in feces at different time points of infection. The shedding of *Brucella* through the feces suggests that there must be another source of infection. It has been shown that *Brucella* can remain viable in manure for up to 2 months under favorable environmental conditions. These results are important because manure contaminated with *Brucella* can originate outbreaks, yet scarce information is available about the risk it presents daily, and the consumption of contaminated food remains the main source of infection in humans [17,18,19].

Altogether, our findings reinforce the importance of having a good research model, since many questions regarding this infection, such as the route of entry of *Brucella*, its dissemination, and its replication niche, remain unanswered.

## 5. Conclusions

The results of this work show that a selective agar medium is not adequate for the direct isolation of *Brucella* from orally inoculated BALB/c mice when using low doses of the *Brucella*. We demonstrated that the selective culture broth allows the recovery and isolation of *Brucella* from BALB/c mice inoculated orally if low doses of the bacteria are employed. These results introduce the possibility of mimicking natural infection with lower doses, thus making the mouse model more adequate and relatable and bringing science closer to a better understanding of brucellosis.

## Figures and Tables

**Table 1:** Growth of *B. abortus* 2308 in selective *Brucella* medium, from Peyer’s patches (PPs), mesenteric lymph nodes (MLNs), and spleens of three mice infected with different doses of bacteria.

Dose (CFUs)	Tissue								
	PPs			MLNs			Spleen		
1 × 10^6^	ND	ND	ND	ND	ND	ND	ND	ND	ND
5 × 10^6^	+	+	+	+	+	+	+	+	+
10 × 10^6^	+	+	+	+	+	+	+	+	+

(+) Bacterial growth; (ND) not detected.

**Table 2:** Detection of *B. abortus* 2308 by direct plate count, from Peyer’s patches (PPs), mesenteric lymph nodes (MLNs), and spleens of three mice orally infected with 5 × 10^6^ CFUs.

**Tissue**	**Time Postinfection**											
	**1 h**			**2 h**			**48 h**			**72 h**		
PP	3.33 × 10^2^ CFU/organ	ND	ND	ND	1.2 × 10^3^	ND	ND	ND	ND	ND	ND	ND
MLNs	ND	ND	ND	ND	ND	ND	ND	ND	ND	ND	ND	ND
Spleen	ND	ND	ND	ND	ND	ND	ND	ND	ND	ND	ND	ND
**Tissue**	**Time Postinfection**											
	**7 d**			**4 Weeks**						**5 Weeks**		
PP	ND	ND	ND	ND		2.6 × 10^3^ CFU/organ		ND		ND	ND	ND
MLNs	ND	UC	ND	5.1 × 10^6^ CFU/organ		ND		ND		ND	ND	ND
Spleen	ND	ND	ND	ND		ND		UC		ND	UC	ND

(ND) Not detected; (UC) uncountable.

**Table 3:** Growth of *B. abortus* 2308 in selective broth for *Brucella*, from Peyer’s patches (PPs), mesenteric lymph nodes (MLNs), spleens, and feces of mice orally infected with 5 × 10^6^ CFUs.

Tissue	Time Postinfection																				
	1 h			2 h			48 h			72 h			7 d			4 Week			5 Week		
PP	+	+	+	+	+	+	+	+	+	+	+	+	ND	ND	ND	+	+	+	ND	ND	ND
MLN	+	+	+	+	+	+	+	+	+	+	+	+	+	+	+	+	+	+	+	+	ND
Spleen	ND	ND	ND	ND	ND	ND	ND	ND	ND	+	+	+	ND	ND	ND	+	+	+	+	+	+
Feces	ND	ND	ND	+	+	+	+	+	+	+	+	ND	ND	ND	ND	+	ND	+	+	+	+

(+) Bacterial growth; (ND) not detected.

## Data Availability

The data presented in this study are available on request from the corresponding author. The data are not publicly available due to some manuscripts in preparation that are closely related to the results included in this paper.

## Abbreviations

The following abbreviations are used in this manuscript:

CFUs	Colony-forming units
MLNs	Mesenteric lymph nodes
PPs	Peyer’s patches
TSA	Trypticase soy agar
